# Temporomandibular disorders, bite force and osseous changes of the temporomandibular joints in patients with hypermobile Ehlers‐Danlos syndrome compared to a healthy control group

**DOI:** 10.1111/joor.13348

**Published:** 2022-06-30

**Authors:** Karen Bech, Frederikke Maria Fogh, Eva Fejerskov Lauridsen, Liselotte Sonnesen

**Affiliations:** ^1^ Section of Orthodontics, Department of Odontology, Faculty of Health and Medical Sciences University of Copenhagen Copenhagen Denmark; ^2^ Resource Center for Rare Oral Diseases, Copenhagen University Hospital Rigshospitalet Copenhagen Denmark

**Keywords:** bite force, CBCT, Ehlers‐Danlos syndrome, hypermobile subtype, temporomandibular disorders

## Abstract

**Background:**

Ehlers‐Danlos syndrome (EDS) is a hereditary disorder that affects the connective tissue and collagen structures in the body characterised by joint hypermobility, skin hyperextensibility and tissue fragility.

**Objective:**

The aim was to investigate temporomandibular disorders (TMD), bite force, teeth in occlusal contact and osseous changes of the temporomandibular joints (TMJs) in 26 patients with hypermobile EDS (hEDS), differentiated by a genetic test, compared to 39 healthy controls.

**Methods:**

Clinical examination according to Diagnostic Criteria for Temporomandibular Disorders (DC/TMD), radiological examinations of the TMJs by cone‐beam‐computed tomographic (CBCT) scans, registration of bite force and teeth in occlusal contact was performed. Statistical analyses included Fisher's Exact Test, multiple logistic and linear regression models adjusted for age, gender and Body Mass Index (BMI).

**Results:**

Single symptoms and signs of TMD occurred significantly more often in hEDS (*p* = .002; *p* = .001; *p* = .003; *p* = <.0001; *p* = .012) and maximum mouth opening was significantly smaller in hEDS compared to controls (*p* = <.0001). The DC/TMD diagnosis myalgia, myofascial pain with referral, arthralgia, headache attributed to TMD, disc displacement disorders and degenerative joint disease occurred significantly more often in hEDS compared to controls (*p* = .000; *p* = .008; *p* = .003; *p* = .000; *p* = <.0001; *p* = .010, respectively). No significant differences were found in bite force and in teeth in occlusal contact between the groups (*p* > .05). On CBCT of the TMJs, subcortical sclerosis occurred significantly more often in hEDS compared to controls (*p* = .005).

**Conclusion:**

Symptoms and signs of TMD and osseous changes of the TMJs occurred significantly more often in hEDS. Bite force and teeth in occlusal contact were comparable to controls.

## Graphical abstract

Ehlers‐Danlos syndrome (EDS) is a hereditary disorder that affects the connective tissue and collagen structures in the body. Symptoms and signs of temporomandibular disorders (TMD) and osseous changes of the temporomandibular joints (TMJs) occurred significantly more often in hypermobile EDS (hEDS). Bite force and teeth in occlusal contact were comparable to controls.

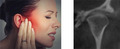

## 1 BACKGROUND

The Ehlers‐Danlos syndrome (EDS) is a clinically and genetically heterogeneous group of heritable connective tissue disorders characterised by joint hypermobility, skin hyperextensibility and tissue fragility.^1^ The estimated prevalence of EDS is 1/5000 and the hEDS is the most frequent.^2^ EDS is caused by mutations in genes involved in collagen structure and/or biosynthesis, but the underlying pathophysiological mechanism is still not fully understood. ^1^ The clinical and genetic heterogeneity of the EDS has long been recognised, and currently EDS is classified into thirteen subtypes according to the 2017 International Classification of the Ehlers‐Danlos Syndromes.^1^ The definite diagnosis for all subtypes, except hEDS with unknown genetic aetiology, depends on molecular confirmation with identification of causative variants in the respective genes owing to the genetic heterogeneity and phenotypic variability of the EDS subtypes together with clinical similarities of EDS and other hereditary connective tissue disorders.^1^ hEDS, which is considered the least severe subtype of EDS, remains a clinical diagnosis, which relies on a number of specific clinical signs such as, e.g., the presence of generalised joint hypermobility, mild skin hyperextensibility and/or smoot velvety skin, and atrophic scarring as well as exclusion of other types of EDS and other heritable and acquired connective tissue disorders, including autoimmune rheumatologic conditions.^1^, ^3^

Variations of oral manifestations and temporomandibular disorders (TMD) have previously been reported in EDS in general where subgroups of EDS were mixed and include deviations in the oral mucosa, lack of lingual and labial frenula, early onset of periodontitis, accelerated tooth movement, deviations in the dentition, orofacial pain, symptoms and signs of TMD.^4^, ^5^, ^6^, ^7^, ^8^ In a group of well‐diagnosed hEDS resistance to local anaesthesia, tooth extraction complications, poor oral hygiene, larger distance between cement‐enamel‐junction and marginal bone level and small crown heights have been found in comparison to healthy controls.^9^ However, only few studies on EDS/inherited connective tissue disorders and symptoms and sign of TMD have previously been performed^8^, ^10^, ^11^, ^12^ and the majority of the studies have mixed the EDS subgroups. Only one study has previously evaluated symptoms and signs of TMD in hEDS patients, but the study was based on only 14 hEDS patients.^10^ As EDS is a clinically and genetically heterogeneous group^1^ it is important to investigate a single well‐diagnosed EDS subgroup and not a mixed EDS group.

Furthermore, the osseous component of the temporomandibular joints (TMJs) may be affected by the underlining connective tissue disorders in hEDS as bone is composed of specialised connective tissue with mineralisation of the extracellular matrix.^13^ Osseous changes in the TMJs have not previously been reported in hEDS.

In addition, the bite force may also be different in hEDS patients. The magnitude of the bite force is dependent on many factors including pain^14^, ^15^, ^16^ and as orofacial pain has been reported in EDS^5^, ^8^ this may influence the magnitude of the bite force in hEDS. Bite force has not previously been reported in hEDS.

As previous studies have found that Body Mass Index (BMI) may influence the masticatory muscles, the TMJs and the bite force,^17^, ^18^, ^19^, ^20^ the analysis of the present study included not only adjustment for age and gender but also for BMI.

The aim of the present study was to assess symptoms and signs of TMD, bite force, teeth in occlusal contact and osseous changes of the TMJs in patients with hEDS, and to compare these findings with a healthy control group.

## 2 MATERIALS AND METHOD

The study followed the guidelines of the Declaration of Helsinki and was approved by the Danish National Committee on Health Research Ethics (Protocol H‐17015290) and the Danish Data Protection Agency (SUND‐2017‐28). The examinations included clinical and radiographic examinations which were recorded at the Department of Odontology, Orthodontics, University of Copenhagen and at the Resource Center for Rare Oral Diseases, Copenhagen University Hospital, Rigshospitalet. In addition, a genetic test was performed on the hEDS patients to exclude other subtypes of EDS.

### 2.1 Participants

Fifty‐one patients with EDS were referred to the Resource Center for Rare Oral Diseases, Copenhagen University Hospital, Rigshospitalet, in the period 2012–2018. Inclusion criteria for hEDS patients were: clinical hEDS diagnosed by a Rheumatologist according to the Villefranche classification,^21^ differentiated by a genetic test to exclude other subtypes of EDS and other heritable and acquired connective tissue disorders, including autoimmune rheumatologic conditions were also excluded. The hEDS ranging from 20 to 50 years, and informed consent given.

The controls were healthy dental students and employees at the Department of Odontology, University of Copenhagen. Inclusion criteria for the controls: no known diseases or syndromes, within the age range from 20 to 52 years, dental students or employees at the Department of Odontology, neutral occlusion, no previous orthodontic treatment, and informed consent given. The genetic test was performed on all hEDS patients using a saliva sample mixed with residue from a mucosa scrape. The sample was tested for mutations in genes associated with other EDS subtypes: COL3A1, COL5A1, COL5A2, COL1A1, COL1A2, PLOD1 and CHST14 in preparation for confirming and excluding EDS subtypes other than hEDS.^1^

A total of 65 participants, 26 hEDS and 39 controls, were included in the study. The 26 hEDS consisted of 4 men and 22 women, mean age 34.5 ± 10.1 years (age range: 20–50), mean BMI 26.5 ± 6.0 (BMI range 17.2–36.6), mean horizontal maxillary overjet 3.77 ± 1.86 mm (range: 1–8 mm) and mean vertical overbite 3.19 ± 1.81 mm (range 1–7 mm). The 39 controls consisted of 11 men and 28 women, mean age 31.8 ± 10.0 years (age range: 20–52), mean BMI 23.5 ± 3.3 (BMI range 18.5–33.7), mean horizontal maxillary overjet 2.46 ± 0.76 mm (range 1–4 mm) and mean vertical overbite 2.54 ± 0.97 mm (range 1–4 mm). All participants were clinically examined using standardised principles. It was not possible to blind the clinical examination. However, all registrations from the clinical examination were blinded and, consequently, the statistical analysis was blinded as well.

Power calculation was performed prior to undertaking the study on the following results of previous studies: It has previously been reported that TMD occurred in 10% in healthy adults^22^ and in 40–100% in patients with EDS.^5^ Under the assumption that TMD occurs in 50% in patients with EDS, at least 17 subjects in each group were required to have sufficient power (80%) to identify statistically significant differences at the 5% level of significance. Thus, the sample size is sufficient in the present study.

### 2.2 Clinical examination

The clinical examination included assessment of symptoms and signs of TMD, registration of bite force and teeth in occlusal contact and was performed by an experienced examiner certified in DC/TMD (LS). The presence of TMD was assessed according to DC/TMD examination form (Axis I).^23^ Single symptoms and signs were recorded, and left and right side recordings were pooled. Ten diagnoses of TMD were evaluated according to DC/TMD examination form (Axis I): myalgia, myofascial pain with referral, arthralgia, headache attributed to TMD, four types of disc displacement disorders, degenerative joint disease and dislocation.^23^

The unilateral bite force was measured at the first mandibular molars on each side during maximal clinching by means of a miniature pressure transducer.^24^ The bite force was measured unilaterally two times in the right side and then two times in the left side as stored peak values during maximum effort and determined as the average of the four measurements.^14^

The number of teeth in contact in the intercuspal position (TOC) was assessed according to the standard method^14^ in the mouth from the ability to hold a plastic strip, 0.05 mm thick and 6 mm wide (Hawe Transparent Strips No. 690, straight®), between the teeth against a strong pull when the patient's teeth were firmly closed.^14^ TOC was then registered on an occlusogram and counted.^14^

BMI was calculated by the standard formula: weight/height^2^. The weight of each participant was measured in kilograms and the height was measured in metres without the participant wearing shoes.

### 2.3 Radiographic examination

The radiographic examination of the TMJs involved a cone beam computed tomography (CBCT) scan, which were recorded at the Cephalometric laboratory, Department of Odontology, University of Copenhagen, obtained in a ProMax ® (3D Max Sensorhead, sensor type: 2520D, Planmeca Oy, Helsinki, 2012). Individual cross‐sections were produced with sagittal sections perpendicular and coronal images parallel to the mediolateral long axis of the condyle. The section thickness was 2 mm for the sagittal and coronal sections. Twelve sections were used for the sagittal plane and 10 for the coronal plane. The images were saved in the program (Romexis software ®, Planmeca Company, Helsinki) and analysed (KB and FMF) after training and calibration with an experienced examiner (LS). If disagreement occurred between the observations the findings were discussed until agreement. According to Ahmad et al.,^25^ osseous changes such as deviation in the relative size of the condyle, articular surface flattening, localised subcortical sclerosis, subcortical cysts, surface erosion, osteophytes and generalised sclerosis were assessed for the condyle, fossa and eminence.

### 2.4 Reliability

The reliability of the clinical examination has previously been assessed as good to excellent,^26^, ^27^ and the method error for the bite force has previously been assessed, s (*i*) = 22.1 N. The interobserver agreement for the CBCTs was calculated on 25 randomly selected CBCTs. The registrations and measurements were repeated after 2 weeks assessed by Kappa. ^28^ The interobserver agreement was good to excellent (κ = 0.75–1).

### 2.5 Statistical analysis

Statistical analyses including the power calculation were performed using SPSS IBM version 25.0, and the level of significance was set to 5%. The categorical data were analysed using multiple logistic regression adjusted for age, gender and BMI. For categorical data that occurred only in one of the groups, these variables were analysed using Fisher's exact test. Q‐Q plots confirmed the normal distribution of the continuous data, which were analysed using multiple linear regression adjusted for age, gender and BMI. *p*‐values corrected for multiple testing comparing groups have been performed using the False Discovery Rate (FDR)^29^ calculated with PROC MULTEST, SAS, version 9.4.

## 3 RESULTS

No statistical significant differences in age, gender, BMI and vertical overbite were found between the hEDS and controls. The horizontal maxillary overjet was statistically significantly larger in the hEDS compared to the controls (*p* < .01).

### 3.1 Clinical examination

Single symptoms and signs of TMD occurred significantly more often in hEDS compared to controls: orofacial pain (*p* < .0001), headache (*p* = .002), pain on opening movements (*p* = .001), pain on lateral (*p* < .000) and protrusive movements (*p* = .031), pain with palpation of masseter (*p* = .003), temporal muscles (*p* < .0001) and TMJs (*p* < .0001), crepitation with jaw movements (*p* < .0001) and joint locking (*p* = .008) (Table 1). Pain‐free mouth opening (*p* < .0001) and maximum mouth opening (*p* = .013) were significantly smaller in the hEDS compared to controls (Table 2). No significant differences were found in lateral movements of the jaws, protrusion, bite force and TOC between the groups (Table 2). The diagnosis according to DC/TMD occurred significantly more often in hEDS compared to controls: myalgia (*p* < .000), myofascial pain with referral (*p* = .008), arthralgia (*p* = .003), headache attributed to TMD (*p* < .000), disc displacement disorder (*p* < .0001) and degenerative joint disease (*p* = .01) (Table 3).

**TABLE 1:** Pain and TMJ noises (Categorical clinical variables). A comparison between 26 patients with hEDS and 39 controls adjusted for gender, age and BMI

Pain and sounds from TMJ								
	hEDS (*N*, %)		Control (*N*, %)		Independent variable (reference)	*p*‐value	OR	95% CI, OR
	Yes	No	Yes	No				
Facial pain	23 (88.5%)	3 (11.5%)	3 (7.7%)	36 (92.3%)	Group (hEDS)	<0.0001*** **(0.0002)**	80.960	14.284;458.862
					Gender (male)	0.770	0.715	.075; 6.798
					Age	0.836	1.010	.919; 1.111
					BMI	0.712	1.037	.856; 1.256
Headache	23 (88.5%)	3 (11.5%)	16 (41%)	23 (59%)	Group (hEDS)	0.002** **(0.003)**	9.120	2.260;36.795
					Gender (male)	0.363	0.478	0.097; 2.345
					Age	0.949	1.002	0.939; 1.069
					BMI	0.117	1.108	0.975; 1.259
Pain at maximum opening movements	15 (57.7%)	11 (42.3%)	4 (10.3%)	35 (89.7%)	Group (hEDS)	0.001** **(0.002)**	9.523	2.401; 37.777
					Gender (male)	0.756	0.781	0.164; 3.714
					Age	0.608	1.018	0.952; 1.088
					BMI	0.265	1.083	0.942; 1.245
Pain at lateral movements	10 (38.5%)	16 (61.5%)	0 (0%)	39 (100%)	Group # (hEDS)	0.000*** **(0.0002)**		
Pain at protrusive movements	9 (34.6%)	17 (65.4%)	1 (2.6%)	38 (97.4%)	Group (hEDS)	0.031* **(0.034)**	13.306	1.262; 140.274
					Gender (male)	0.770	0.800	0.178; 3.586
					Age	0.671	1.014	0.951; 1.082
					BMI	0.440	1.057	0.918; 1.218
Muscle pain with palpation – Temporalis	23 (88.5%)	3 (11.5%)	10 (25.6%)	29 (74.4%)	Group (hEDS)	<0.0001*** **(0.0002)**	21.067	4.572; 97.073
					Gender (male)	0.989	0.988	0.168; 5.805
					Age	0.377	1.033	0.961; 1.110
					BMI	0.638	1.034	0.898; 1.191
Muscle pain with palpation ‐ Masseter	25 (96.2%)	1 (3.8%)	10 (25.6%)	29 (74.4%)	Group (hEDS)	0.003** **(0.005)**	26.284	3.151; 219.239
					Gender (male)	0.550	0.601	0.113; 3.185
					Age	0.796	1.009	0.943; 1.079
					BMI	0.184	1.092	0.959; 1.243
Muscle pain with palpation ‐ TMJ	17 (65.4%)	9 (34.6%)	4 (10.3%)	35 (89.7%)	Group (hEDS)	<0.0001*** **(0.0002)**	13.909	3.535; 54.726
					Gender (male)	0.329	0.426	0.077; 2.364
					Age	0.912	1.004	0.937; 1.075
					BMI	0.271	1.087	0.937; 1.260
Click at jaw movements	17 (65.4%)	9 (34.6%)	18 (46.2%)	21 (53.8%)	Group (hEDS)	0.111 (0.11)	2.517	0.810; 7.823
					Gender (male)	0.416	0.539	0.112; 2.389
					Age	0.889	1.004	0.947; 1.065
					BMI	0.017*	1.165	1.028; 1.318
Crepitus at jaw movements	15 (57.5%)	11 (42.3%)	6 (15.4%)	33 (84.6%)	Group (hEDS)	<0.0001*** **(0.0002)**	15.748	3.470; 71.460
					Gender (male)	0.109	0.236	0.040; 1.378
					Age	0.904	0.996	0.929; 1.067
					BMI	0.009**	1.230	1.052; 1.437
Click and crepitus at jaw movements	23 (88.5%)	3 (11.5%)	23 (59%)	16 (41%)	Group (hEDS)	0.012* **(0.014)**	6.803	1.530; 30.247
					Gender (male)	0.350	0.473	0.098; 2.273
					Age	0.896	1.004	0.944; 1.068
					BMI	0.016*	1.173	1.030; 1.335
Joint locking	5 (19.2%)	21 (80.8%)	0 (0%)	39 (100%)	Group # (hEDS)	0.008** **(0.011)**		

*Note*: The *p*‐values marked in bold are significant *p*‐values corrected for multiple testing comparing groups.

Abbreviations: CI, Confidence interval; OR, Odds ratio.

*p < .05; **p < .01; ***p < .001; #Fisher's Exact Test was performed.

**TABLE 2:** Bite force, teeth in occlusal contact and jaw movements (continuous clinical variables). A comparison between 26 patients with hEDS and 39 controls adjusted for gender, age and BMI

Continuous clinical variables						
Dependant variable			Independent variable (reference)	*p*‐value	B	95% CI B
	hEDS	Controls				
Bite force (N)	Mean: 263.96	Mean: 340.7	Group (hEDS)	0.978 (0.98)	−1.319	−95.740; 93.102
	SD: 166.66	SD: 160.36	Gender (male)	0.011*	−134.881	−237.261; −32.502
	Min: 26.75	Min: 121.5	Age	0.061	−5.617	−11.511; 0.276
	Max: 659	Max: 693.25	BMI	0.774	1.469	8.788; 11.726
Teeth in occlusal contact	Mean: 8.65	Mean: 10.31	Group (hEDS)	0.198 (0.35)	0.735	−0.395; 1.865
	SD: 2.70	SD: 1.54	Gender (male)	0.891	0.096	−1.296; 1.488
	Min: 1	Min: 8	Age	0.676	−0.012	−0.71; 0.046
	Max: 16	Max: 13	BMI	0.219	−0.078	−0.202; 0.047
Pain‐free opening	Mean: 38.62	Mean: 52.95	Group (hEDS)	<0.0001*** **(0.0007)**	11.338	6.917; 15.759
	SD: 12.88	SD: 5.63	Gender (male)	0.016*	−6.578	−11.884; −1.271
	Min: 15	Min: 40	Age	0.268	‐0.129	‐0.361; 0.102
	Max: 64	Max: 67	BMI	0.014*	−0.602	−1.077; −0.128
Maximum unassisted opening	Mean: 48.31	Mean: 55.26	Group (hEDS)	0.013* **(0.046)**	4.744	1.054; 8.435
	SD: 9.72	SD: 6	Gender (male)	0.007*	−6.209	−10.639; −1.779
	Min: 20	Min: 40	Age	0.179	−0.131	−0.324; 0.062
	Max: 70	Max: 67	BMI	0.081	−0.352	−0.748; 0.045
Maximum assisted opening	Mean: 49.38	Mean: 56.05	Group (hEDS)	0.022* (0.051)	4.342	0.636; 8.048
	SD: 9.81	SD: 5.96	Gender (male)	0.012*	−5.761	−10.209; −1.312
	Min: 20	Min: 40	Age	0.175	−0.133	−0.327; 0.061
	Max:70	Max: 68	BMI	0.044*	−0.409	−0.807; −0.012
Protrusion	Mean: 6.04	Mean: 6.54	Group (hEDS)	0.785 (0.98)	0.154	−0.973; 1.282
	SD: 2.47	SD: 1.9	Gender (male)	0.584	−0.373	−1.726; 0.980
	Min: 2	Min: 3	Age	0.100	−0.049	−1.08; 0.010
	Max: 12	Max: 11	BMI	0.369	−0.055	−0.176; 0.066
Lateral movements	Mean: 9.98	Mean: 10.33	Group (hEDS)	0.855 (0.098)	0.101	−1.006; 1.208
	SD: 2.36	SD: 2.14	Gender (male)	0.047*	−1.349	−2.678; −0.020
	Min: 4	Min: 6	Age	0.927	−0.003	−0.061; 0.055
	Max: 15	Max: 15	BMI	0.690	−0.024	−0.143; 0.095

*Note*: The *p*‐values marked in bold are significant *p*‐values corrected for multiple testing comparing groups.

Abbreviations: B, parameter estimate; CI, Confidence interval.

*p < .05; **p < .01; ***p < .001.

**TABLE 3:** TMD diagnosis according to DC/TMD examination form (Axis I). A comparison between 26 patients with hEDS and 39 controls adjusted for gender, age and BMI

DC/TMD diagnoses								
	hEDS (*N*, %)		Control (*N*, %)		Independent variable (reference)	*p*‐value	OR	95% CI, OR
	Yes	No	Yes	No				
Myalgia	5 (19.2%)	21 (80.8%)	0 (0%)	39 (100%)	Group # (hEDS)	0.000*** **(0.0003)**		
Myofascial pain with referral pain	5 (19.2%)	21 (80.8%)	0 (0%)	39 (100%)	Group # (hEDS)	0.008** **(0.013)**		
Arthralgia	6 (23.1%)	20 (76.9%)	0 (0%)	39 (100%)	Group # (hEDS)	0.003** **(0.006)**		
Headache attributed to TMD	13 (50%)	13 (50%)	0 (0%)	39 (100%)	Group # (hEDS)	0.000*** **(0.0003)**		
Disc displacement with reduction	4 (15.4%)	22 (84.6%)	13 (33.3%)	26 (66.7%)	Group (hEDS)	0.223(0.22)	0.444	0.120; 1.638
					Gender (male)	0.311	0.477	0.114; 1.996
					Age	0.963	0.999	0.941; 1.059
					BMI	0.030*	1.144	1.013; 1.293
Disc displacement with reduction with limited opening	2 (7.7%)	24 (92.3%)	0 (0%)	39 (100%)	Group # (hEDS)	0.156(0.18)		
Disc displacement without reduction without limited opening	14 (53.8%)	12 (46.2%)	2 (5.1%)	37 (94.9%)	Group (hEDS)	<0.0001*** **(0.0003)**	34.478	5.425; 219.129
					Gender (male)	0.799	0.807	0.154; 4.229
					Age	0.898	0.995	0.924; 1.072
					BMI	0.011*	1.225	1.048; 1.432
Degenerative joint disease	10 (38.5%)	16 (61.5%)	5 (12.8%)	34 (87.2%)	Group (hEDS)	0.010* **(0.013)**	6.685	1.582; 28.240
					Gender (male)	0.164	0.326	0.067; 1.584
					Age	0.724	0.989	0.928; 1.053
					BMI	0.012*	1.194	1.039; 1.372

*Note*: Disc displacement with reduction, with intermittent locking and dislocation were not detected in any of the two groups.

The *p*‐values marked in bold are significant *p*‐values corrected for multiple testing comparing groups.

Abbreviations: CI, Confidence interval; OR, Odds ratio.

*p < .05; **p < .01; ***p < .001; # Fisher's Exact Test was performed.

### 3.2 Radiographic examination

Osseous changes in the TMJs assessed on CBCT occurred significantly more often in hEDS compared to controls: localised subcortical sclerosis (*p* = .005) (Table 4).

**TABLE 4:** Osseous changes of the TMJs (Radiological findings). A comparison between 26 patients with hEDS and 39 controls adjusted for gender, age and BMI

Radiological findings								
	hEDS (*N*, %)		Control (*N*, %)		Independent variable (reference)	*p*‐value	OR	95% CI, OR
	Yes	No	Yes	No				
Condylar hypoplasia	8 (30.8%)	18 (69.2%)	5 (12.8%)	34 (87.2%)	Group (hEDS)	0.100 (0.23)	3.141	0.802; 12.294
					Gender (male)	0.356	0.504	0.118; 2.157
					Age	0.765	1.009	0.950; 1.073
					BMI	0.057	1.126	0.997; 1.273
Articular surface flattening	17 (65.4%)	9 (34.6%)	24 (61.5%)	15 (38.5%)	Group (hEDS)	0.869 (0.87)	1.098	0.359; 3.361
					Gender (male)	0.300	0.469	0.112; 1.965
					Age	0.992	1.000	0.944; 1.060
					BMI	0.036*	1.141	1.009; 1.290
Subcortical sclerosis	15 (57.7%)	11 (42.3%)	10 (25.6%)	29 (74.4%)	Group (hEDS)	0.005* **(0.045)**	5.663	1.674; 19.160
					Gender (male)	0.668	0.712	0.151; 3.354
					Age	0.827	1.008	0.946: 1.072
					BMI	0.016*	1.177	1.030; 1.344
Subcortical cyst	2 (7.7%)	24 (92.3%)	1 (2.6%)	38 (97.4%)	Group (hEDS)	0.322 (0.48)	3.789	0.272; 52.783
					Gender (male)	0.301	0.469	0.112; 1.970
					Age	0.801	0.992	0.933; 1.055
					BMI	0.026*	1.152	1.017; 1.305
Surface erosion	2 (7.7%)	24 (92.3%)	3 (7.7%)	36 (92.3%)	Group (hEDS)	0.844 (0.87)	1.225	0.163; 9.207
					Gender (male)	0.295	0.465	0.111; 1.946
					Age	0.989	1.000	0.942; 1.060
					BMI	0.033*	1.144	1.011; 1.294
Osteophyte	7 (26.9%)	19 (73.1%)	3 (7.7%)	36 (92.3%)	Group (hEDS)	0.014* (0.063)	8.459	1.538; 46.513
					Gender (male)	0.692	0.737	0.162; 3.350
					Age	0.447	1.026	0.961: 1.095
					BMI	0.018*	1.166	1.027; 1.324
Generalised sclerosis	1 (3.8%)	25 (96.2%)	1 (2.6%)	38 (97.4%)	Group (hEDS)	0.611 (0.79)	2.123	0.116; 38.714
					Gender (male)	0.327	0.486	0.115; 2.057
					Age	0.949	1.002	0.945; 1.062
					BMI	0.031*	1.145	1.013; 1.296
Deviation in form of the condyle	7 (26.9%)	19 (73.1%)	3 (7.7%)	36 (92.3%)	Group (hEDS)	0.039* (0.12)	5.890	1.090; 31.839
					Gender (male)	0.211	0.389	0.088; 1.710
					Age	0.502	0.978	0.918; 1.043
					BMI	0.025*	5.890	1.090; 31.839
Fossa/eminence: Subcortical sclerosis	4 (15.4%)	22 (84.6%)	2 (5.1%)	37 (94.9%)	Group (hEDS)	0.192 (0.35)	3.697	0.520; 26.302
					Gender (male)	0.333	0.491	0.116; 2.072
					Age	0.716	0.989	0.930; 1.051
					BMI	0.026*	1.154	1.018; 1.308

*Note*: Condylar hyperplasia, Loose joint body, Bony ankylosis, Fossa/eminence: Articular surface flattening and Fossa/eminence: Surface erosion were not detected in any of the two groups.

The *p*‐value marked in bold is significant *p*‐value corrected for multiple testing comparing groups.

Abbreviations: CI, Confidence interval; OR, Odds ratio.

*p < .05; **p < .01; ***p < .001.

## 4 DISCUSSION

The aim of the present study was to compare TMD, bite force, TOC and osseous changes in the TMJs in hEDS patients with healthy controls. Only one studie has investigated TMD in a subgroup of 14 hEDS patients,^10^ and to our knowledge, bite force, TOC and osseous changes of the TMJ evaluated on CBCTs in hEDS patients compared to controls have not previously been described. In the present study, a genetic test was performed to improve the homogeneity of the sample, and accordingly, the diagnostic significance of the results. Furthermore, the participants were diagnosed by a rheumatologist based on the Villefrance classificaion^21^ and other heritable and acquired connective tissue disorders, including autoimmune rheumatologic conditions were excluded. The clinical presentation of Osteogenesis Imperfecta (OI) type I may also include loose joints and easy bruising, and it may therefore be possible that mild cases of OI type I could be misinterpreted as hEDS. However, the participants in the present study did not show other clinical signs of OI type I such as history of early bone fractures and blue sclera. Therefore, OI type I was not included in the genetic analysis. The clinical and radiological examination was performed by standard validated procedures and protocols,^14^, ^23^, ^24^, ^25^ but it was not possible to blind the clinical part of the examinations. However, all registrations from the examinations were blinded and, consequently, the statistical analysis was blinded as well.

### 4.1 Clinical examination

The structure of the collagen and its function are altered in all subtypes of EDS ^1,^ and may cause symptoms in the different orofacial systems. In the TMJs, the fibrocartilage structures, supporting ligaments, the disc and the retrodiscal tissues are composed mainly of collagen.^30^, ^31^ In the present study, single symptoms and signs of TMD such as orofacial pain, pain on jaw movements and pain with muscle and TMJ palpation occurred significantly more often in hEDS patients compared to controls. This is in agreement with previous studies where it was found that the muscular‐skeletal pain in general was often reported in all subgroups of EDS patients.^1^, ^32^, ^33^ Furthermore, crepitation with jaw movements and joint locking occurred significantly more often in hEDS patients compared to controls. It has previously been found that joint sounds and locking of the jaw may occur in hypermobile joints^34^, ^35^ due to dislocation of the cartilaginous disc once the TMJ is hyperextended resulting in pain, bony destruction and restricted mandibular mobility.^5^, ^36^, ^37^ Thus, this may also explain the surprising finding of restricted mouth opening, and the significant pain on jaw movements in hEDS compared to controls in the present study. No significant differences were found in clicking of the joints between the groups in the present study. This may be due to the fluctuation of clicking of the joints in the general population, which may range between 10 and 25%, rather than the underlying connective tissue disorder in the present study.^38^, ^39^

In the present study, the diagnosis according to DC/TMD, myalgia, myofascial pain with referral, arthralgia, headache attributed to TMD, disc displacement disorder and degenerative joint disease occurred significantly more often in hEDS compared to controls. Only one study has evaluated TMD according to DC/TMD protocol, though in a mixed group of EDS patients, and no control group was included in the study.^12^ The study reported that the most common diagnosis in EDS was arthralgia, myalgia, disc displacement disorder, subluxation and headache attributed to TMD. It has previously been reported that the majority of patients with EDS had a combination of myofascial pain, internal joint derangement and arthralgia of one or both TMJs,^11^ which is in agreement with the present study on hEDS patients.

In the present study, the horizontal maxillary overjet was significantly larger in hEDS compared to the controls. It is unknown if the large horizontal maxillary overjet in the hEDS was due to a dentoalveolar or craniofacial skeletal discrepancy or a combination as this was not the aim of the present study. Furthermore, the large horizontal maxillary overjet in hEDS was not found to be in combination with fewer teeth in occlusal contact or smaller bite force when compared to the controls. Therefore, and because the occurrence of TMD is influenced by multiple factors^22^, ^26^, ^27^, ^40^ the significant larger horizontal maxillary overjet in the hEDS is not considered a factor in the different occurrence of TMD between the two groups.

In the present study, no significant differences in bite force and TOC were found between hEDS patients and controls. Bite force and TOC have not previously been reported in hEDS. It was expected to find a significant difference in bite force and TOC in the present study, as hEDS is a connective tissue disorder which may cause decreased muscle function in general.^41^ On the other hand, the magnitude of the bite force is dependent on many factors such as pain, TOC, dental occlusion and craniofacial morphology besides age, gender and BMI, which the bite force was adjusted for in the present study.^14^, ^15^, ^16^, ^17^ No significant difference in age, gender, BMI and TOC between the groups was found in the present study, which may indicate that these factors may be more important for the magnitude of the bite force than the underlying connective tissue disorder.

### 4.2 Radiographic examination

Osseous changes in the TMJs assessed on CBCT in hEDS have not previously been reported in the literature. In the present study, localised subcortical sclerosis occurred significantly more often in hEDS compared to controls. This may be explained by the underlying connective tissue disorder in hEDS as bone is composed of specialised connective tissue with mineralisation of the extracellular matrix.^13^ On the other hand, there is an individual variation in the morphology of the condyle depending on, e.g., genetics, function, overload, dental occlusion, trauma, other developmental disorders and age.^42^, ^43^, ^44^ Furthermore, there may not always be a direct relationship between osseous changes of the TMJs and symptoms of TMD,^45^ and some osseous changes may indicate a non‐pathological remodelling of the TMJs due to the continuous adaptive remodelling of the TMJs.^23^, ^46^

## 5 CONCLUSION

Single symptoms and signs of TMD occurred significantly more often in hEDS, and maximum mouth opening was significantly smaller in hEDS compared to controls. The diagnosis myalgia, myofascial pain with referral, arthralgia, headache attributed to TMD, disc displacement disorders and degenerative joint disease occurred significantly more often in hEDS compared to controls. No significant differences were found in bite force and TOC between the groups. On CBCT of the TMJs, subcortical sclerosis occurred significantly more often in hEDS compared to controls.

## AUTHORS' CONTRIBUTION

Karen Bech and Frederikke Maria Fogh: analysis, and interpretation of data for the work; drafting the work; final approval of the version to be published and agreement to be accountable for all aspects of the work in ensuring that questions related to the accuracy or integrity of any part of the work are appropriately investigated and resolved. Eva Fejerskov Lauridsen: contributions to the conception and design of the work and interpretation of data for the work; revising the work critically for important intellectual content; final approval of the version to be published and agreement to be accountable for all aspects of the work in ensuring that questions related to the accuracy or integrity of any part of the work are appropriately investigated and resolved. Liselotte Sonnesen: contributions to the conception and design of the work, acquisition, analysis and interpretation of data for the work; drafting the work and revising the work critically for important intellectual content; final approval of the version to be published and agreement to be accountable for all aspects of the work in ensuring that questions related to the accuracy or integrity of any part of the work are appropriately investigated and resolved.

## CONFLICT OF INTEREST

No conflict of interest declared.

## Data Availability

The data that support the findings of this study are available from the corresponding author upon reasonable request.
